# Osteosarcoma (Osteogenic sarcoma)

**DOI:** 10.1186/1750-1172-2-6

**Published:** 2007-01-23

**Authors:** Piero Picci

**Affiliations:** 1 Rizzoli Orthopaedic Institute, Scientific Institution for Research Hospitalization and Health Care, Bologna, Italy

## Abstract

Osteosarcoma is a primary malignant tumour of the skeleton characterised by the direct formation of immature bone or osteoid tissue by the tumour cells. The classic osteosarcoma is a rare (0.2% of all malignant tumours) highly malignant tumour, with an estimated incidence of 3 cases/million population/year. Osteosarcoma arises predominantly in the long bones and rarely in the soft tissues. The age at presentation ranges from 10 to 25 years of age. Plain radiographs, computed tomography, magnetic resonance imaging, angiography and dynamic bone scintigraphy are used for diagnosis, evaluation the extent of tumour involvement and decision of the type of operation and, if necessary, the type of reconstruction. Years ago, all patients with osteosarcoma were treated by amputation but the cure rate was under 10% and almost all patients died within a year from diagnosis. Today, for localised osteosarcoma at onset (80% of cases) treated in specialized bone tumour centres with pre- and postoperative chemotherapy associated with surgery, the percentage of patients cured varies between 60% and 70%. Surgery is conservative (limb salvage) in more than 90% of patients. Prognosis is more severe (cure rate about 30%) for tumours located in the axial skeleton and in patients with metastasis at onset.

## Disease name and synonyms

Osteosarcoma

Osteogenic sarcoma

## Definition and diagnostic criteria

Osteosarcoma is a primary malignant tumour of the skeleton characterised by the direct formation of immature bone or osteoid tissue by the tumour cells. More rarely osteosarcoma may arise in the soft tissue.

World Health Organization (WHO) histologic classification of bone tumours divides osteosarcomas into central and surface tumours, and recognises a number of subtypes within each group [1]. This paper refers only to the conventional central high grade primary osteosarcoma of bone, which represents about 90% of all cases of osteosarcoma.

### Diagnostic criteria

To confirm diagnosis, a biopsy is always required. Biopsy material should be obtained by the use of either a large-core tissue biopsy or, preferably, by an open biopsy. The use of cytologic or fine-needle aspiration should be avoided as it frequently leads to under-diagnosis or incorrect diagnosis.

It is important to place the biopsy tract in an area where it can be totally excised, if the patient will be successively treated by limb salvage. When a malignant bone tumour is suspected, it is preferable the initial biopsy to be done by the surgeon who will do the definitive surgery.

### Stages of osteosarcoma

Once the diagnosis of osteosarcoma has been confirmed, more tests should be done to detect whether cancer cells have spread to other parts of the body.

The currently used Musculoskeletal Tumour Society staging system [2] is reported in Table 1. This system based on:

**Table 1:** Surgical staging of bone sarcomas

**Stage**	**Grade**	**Site**	**Metastasis**
IA	Low	Intracompartmental	No
IB	Low	Extracompartimental	No
IIA	High	Intracompartimental	No
IIB	High	Extracompartimental	No
III	Any	Any	Regional or distant

• tumour grade (I = low grade; II = high grade);

• tumour extension (A = intraosseous involvement only; B = intra- and extraossseous extension);

• presence of distant metastases (III).

Patients with localised high grade osteosarcoma may have stage IIA or IIB. The presence of metastatic disease, regardless the extent of the primary lesion, represents a stage III disease. A bone scan should be done to rule out bone metastases. Computed tomography scan of the chest should also be performed to rule out pulmonary metastases.

## Epidemiology

Classic osteosarcoma represents approximately 15% of all biopsy-analysed primary bone tumours. Among primary malignant bone tumours, it ranks second in frequency after multiple myelomas. The incidence of classic osteosarcoma is 3 cases/million population/year. It represents 0.2% of all malignant tumours [3].

In about 75% of cases, patients with osteosarcoma are between 15–25 years of age. Male are more frequently affected than female (ration 1.5:1). Osteosarcoma rarely occurs in patients younger than 6 years or older than 60 years. Tumours observed in older age usually develop secondary to Paget's disease, radiation or dedifferentiated chondrosarcomas.

In general, 80% to 90% of osteosarcomas occur in the long tubular bones. The axial skeleton is rarely affected, more frequently in adults than in children and adolescents. Femur, tibia and humerus account for about 85% of extremity tumours, while less than 1% are found in hands and feet bones. In the long bones, osteosarcoma usually originate in the metaphysis. Tumours originating in the midshaft are uncommon and these originating in the epiphysis are very rare.

## Clinical description

Most patients who present with osteogenic sarcoma of the extremities complain of pain prior to soft tissue swelling. This is true of any primary bone tumour, because stretching of the periosteum usually causes pain before the tumour is discernible. Pain could also result from weakening of the bone with development of minute stress fractures. Development of sudden and severe pain heralds gross pathologic fracture, which is an uncommon finding in adult patients. Up to 15% of paediatric patients present a pathological fracture.

The second most common complaint is swelling, which is related to the soft tissue mass. Although about 90% of osteosarcoma show soft tissue extension, a few patients complain of swelling.

Systemic symptoms as weight loss, pallor, fever, anorexia are very uncommon.

## Etiology

Etiology of osteosarcoma is unknown. A viral origin was suggested by the evidence that bone sarcomas can be induced in selected animals by viruses or cell-free extracts of human osteosarcomas [4]. The only environmental agent known to cause osteosarcoma in human is ionising radiation [4]. Radiation is implicated in approximately 2% of osteosarcomas. An increased incidence of radio-induced osteosarcoma is likely to be seen with a longer survival after primary irradiation.

Several families have been described with multiple members who developed osteosarcoma, suggesting genetic predisposition to this tumour [5]. So far, the strongest genetic predisposition is found in patients with hereditary retinoblastoma. In patients with retinoblastoma, osteosarcoma occurs 500 times more frequently than in the general population [6]. Screening large series of children with osteosarcoma revealed that approximately 3% to 4% carried a constitutional germline mutation in p53 [7]. The majority of cases with germline p53 mutations represent patients with a family history suggestive of Li-Fraumeni syndrome.

## Diagnostic methods

Characteristically, plain radiographs of the involved bone show a mixed sclerotic or lytic lesion in the affected area. The tumour erodes through the cortex, causing elevation of periosteum and often produces a significant soft tissue swelling. It is important to remember that periosteal elevation in an apparent bone lesion is an indication for biopsy.

Computed tomography (CT), magnetic resonance imaging (MRI), angiography and dynamic bone scintigraphy are also important, especially to evaluate the extent of tumour involvement. These are of great help to the surgeon to decide the type of operation (amputation, limb salvage or rotationplasty) and, if necessary, the type of reconstruction. An elevated level of serum alkaline phosphatase, which is found in more than 40% of patients, is also a valuable diagnostic parameter. However, due to the difficulties in general standardisation, this parameter may be difficult to interpret in younger patients.

## Differential diagnosis

Diagnosis of osteosarcoma is usually easy. Imaging studies alone, however, may be occasionally misleading. Purely osteolytic osteosarcoma may mimic malignant fibrous histiocytoma, fibrosarcoma or giant cell tumours. Osteosarcoma with diaphyseal location may suggest Ewing's sarcoma or lymphoma [8].

Histologically, osteosarcoma may have to be distinguished from a malignant fibrous histiocytoma or a poorly differentiated fibrosarcoma. Exceptionally, an osteosarcoma histologically mimics an osteoblastoma or an aneurysmal bone cyst [3,8].

## Management including treatment

Patients with high grade osteosarcoma are usually grouped depending on whether the cancer is found in only one part of the body (localised disease) or it has spread to distant tissues or organs (generally lung):

a) Localised osteosarcoma: Cancer cells have not spread beyond the primary bone involved, or nearby tissue in which the cancer began.

b) Metastatic osteosarcoma: At the time of diagnosis, cancers cells have spread from the bone where the cancer began to other parts of the body.

c) Recurrent osteosarcoma: The cancer reappeared in a patient after treatment.

Specific treatment regimens are used for each group of osteosarcoma.

### Localised disease

Radical surgical treatment used alone usually fails in about 85%–90% of patients, due to the high frequency of micro-metastases in high grade osteosarcoma. Dramatic therapeutic improvement achieved in the last 25 years is a result of development of aggressive and efficient combination chemotherapy regimens to fight micro-metastases. Rationale for this approach was that micro-metastatic disease could be more efficiently eliminated when treatment was started early, *i.e*. when the total tumour burden was still small. Thus, modern treatment programmes are typically multimodal, with surgery combined with both pre- and postoperative chemotherapy (neoadjuvant chemotherapy).

As the tumour is radioresistent at standard doses, the radiotherapy plays no significant role in the treatment of osteosarcoma. It can be used, with limited effects, when surgery is not feasible.

Drugs used in treatment of osteosarcoma are high-dose Methotrexate (HDMTX), Cisplatin (CDP), Adriamycin (ADM) and Ifosfamide (IF), generally used in combination. It is not clear if a 4-drugs combination (HDMTX, CDP, ADM, IF) offers advantages over a 3-drugs combination (HDTX, CDP, DM) treatment. In some centres, ADM and CDP are delivered intra-arterially.

Complete surgical resection is crucial for osteosarcoma cure. It may be achieved by amputation, limb salvage (removal of the malignant bone tumour without amputation, and replacement of bones and joints with allografts or prosthetic devices) or rotationplasty [9]. Today, about 80%–90% of patients are treated with limb salvage, which should be performed by an orthopaedic surgeon with a good experience in the treatment of bone tumours. In fact, if adequate surgical margins are not achieved, the rate of local recurrence is very high (about 25%); in addition, the local recurrence has a very bad prognostic significance [10-12]. Functional results of non demolitive surgery in these patients improved dramatically in the last ten years. This improvement reflects the interest of the orthopaedic oncologists in the high survival rates achieved through the use of chemotherapy.

### Metastatic disease

If CT scan of the chest shows presence of pulmonary nodules, they should be resected together with the primary tumour or at least successively, stopping all therapy. This should be done even if, in our experience, solitary nodules are non metastatic lesions but pseudometastasis in about 25% of cases [13]. However, in a young patient with a classic osteogenic sarcoma the diagnosis of pseudometastases should be always histologically confirmed, following the removal of the lesion from the lung. Even if the nodules disappear completely after preoperative chemotherapy, the patient should undergo a thoracotomy. Microscopic small deposits of residual tumour may exist, and they will most probably recur in the original CT-positive areas if the patients does not have a thoracotomy; removal of the residual disease, which can be readily palpated as a small "grain of sand" must be performed by thoracic surgeon. The pre- and postoperative chemotherapeutic treatment should be the same as that for patients with localised disease. The probability of cure for the uncommon cases in which metastatic disease at presentation is located outside the lung is less than 5% [14,15].

### Recurrent osteosarcoma

Recurrence of osteosarcoma in more than 90% of patients is located in the lung. Patients with recurrent disease, when possible, should always be treated by surgery. Ability (and possibility) of achieving a complete resection of metastatic lesions is the most relevant prognostic factor, with (at first relapse) a 5-year survival rate of 20% to 45% following complete resection of metastatic pulmonary lesions, and 10% to 15% following complete resection of metastases in other sites [14,15]. Factors associated with better outcome in recurrent osteosarcoma include solitary pulmonary nodules, a long interval from initial diagnosis and achievement of a second complete remission. The role of a second-line chemotherapy after treatment of metastatic disease is not definitively established.

## Prognostic factors

Combined neoadjuvant treatment gives a cure rate of 60%–70% for patients with nonmetastatic osteosarcoma of the extremities at presentation [16-19] and of about 30% for tumours of the axial skeleton. Metastases at presentation and, in localised tumours, anatomic site (extremity or axial), histological response to preoperative chemotherapy (as measured histologically from the resected specimen), serum levels of alkaline phosphatase and lactate dehydrogenase are the most powerful predictors of survival for patients with osteosarcoma [16-20].
